# Early postoperative outcomes following bariatric surgery in the United States: Are racial disparities improving?

**DOI:** 10.1007/s00464-024-11056-7

**Published:** 2024-07-26

**Authors:** Margaux N. Mustian, Gurudatta Naik, Lauren Wood, Kristen Wong, Richard Stahl, Jayleen Grams, Daniel I. Chu

**Affiliations:** 1 Department of Surgery, Division of Gastrointestinal Surgery, University of Alabama at Birmingham, 1808 7th Avenue South, Boshell Diabetes Building, 5th Floor, Birmingham, AL 35294 USA; 2 Department of Surgery, Birmingham Veterans Affairs Medical Center, Birmingham, AL USA

**Keywords:** Bariatric, Obesity, Disparities, Race, Perioperative outcomes

## Abstract

**Background:**

Bariatric surgery offers effective treatment for morbid obesity and associated medical comorbidities, with excellent short- and long-term outcomes. Although it has been well documented that racial minority bariatric patients have worse outcomes than White patients, it remains unclear whether this recognition has led to improvement. Herein, we assess recent trends in bariatric surgery among Black and White patients and compare early postoperative outcomes by race.

**Methods:**

Primary sleeve gastrectomy (SG) and Roux-en-Y gastric bypass (RYGB) patients from 2015 to 2021 reported to the Metabolic and Bariatric Surgery Accreditation and Quality Improvement Program were studied. Bariatric patients were stratified by race (Black and White) and perioperative outcomes were compared between matched Black and White patients. Primary outcome was 30-day mortality. Secondary outcomes included hospital readmissions, hospital length of stay (LOS), reoperation, and postoperative complications.

**Results:**

Overall, there were 193,071 Black and 645,224 White primary bariatric patients, with a higher volume of SG and RGYB performed among White patients. A total of 219,566 Black and White bariatric patients were matched and included in the case–control. Black patients were found to have higher rates of 30-day mortality (0.02% vs. 0.01%; *p* = 0.03) and readmissions (3.68% vs. 2.65%; *p* < 0.001). There were no significant differences in LOS, reoperations, or overall postoperative complications. However, there was a higher postoperative pulmonary thromboembolism rate (0.16% vs 0.08%; *p* < 0.001).). The differences in perioperative outcomes stratified by race persisted over the study period (Fig. 1).

**Conclusion:**

Black bariatric surgery patients continue to have worse perioperative outcomes compared with their White counterparts. Further work must be done to determine contributing factors in order to effect improvement in outcomes in bariatric surgical care for racial minority patients.

Obesity remains an epidemic in the United States, with greater than 30% of the adult population living with obesity [1]. This national trend has also led to an increase in associated medical comorbidities, such as hypertension, diabetes, and congestive heart failure [2]. Bariatric and metabolic surgery has been established as an effective and durable treatment for obesity and associated medical comorbidities, and it also results in improvement in quality of life [3–9]. Additionally, the safety of bariatric surgery has continued to improve over the last several decades [10].

In the United States, the prevalence of obesity remains higher among Black patients, compared with their White and Asian counterparts [11]. Despite the high prevalence of obesity, bariatric surgery remains underutilized throughout the nation but particularly among Black patients [12]. Furthermore, racial disparities do exist, with racial minorities having worse perioperative outcomes following bariatric surgery compared with their White counterparts. In a national study from 2015, Sheka et al. demonstrated that Black patients have higher readmissions and longer hospital length of stay (LOS) following laparoscopic Roux-en-Y gastric bypass (RYGB) and sleeve gastrectomy (SG) [13]. Edwards et al. also found two-fold higher 30-day mortality rates for non-Hispanic Black patients undergoing bariatric surgery, compared with non-Hispanic White patients [14].

However, it remains unclear whether recognition of these disparities over the last decade has led to any improvement in outcomes. Therefore, the purpose of this study was to perform a contemporary assessment of perioperative outcomes following bariatric surgery at the national level. We hypothesized that racial disparities for bariatric surgical care persist in the United States and that further work is needed to understand and address these disparities.

## Materials and methods

### Study population

A retrospective study was performed of primary SG and RYGB patients reported to the Metabolic and Bariatric Surgery Accreditation and Quality Improvement Program (MBSAQIP) from January 1, 2015 through December 31, 2021. Data was obtained from the MBSAQIP participant use data file (PUF), based on all programs participating in the MBSAQIP accreditation. This study was approved by the Institutional Review Board.

The bariatric surgery patients identified in the MBSAQIP dataset were then stratified by race (White and Black). Exclusion criteria included: revisional bariatric surgery, bariatric surgery other than SG or RYGB, and patients with missing race/ethnicity or race listed as any other than White or Black race.

### Statistical analyses

Within the overall cohort, descriptive statistics by race (White and Black) were performed over the study period. Patient demographics and characteristics were captured including age, body mass index (BMI) at time of operation, sex (male/female), operation performed (SG or RYGB), American Society of Anesthesia (ASA) classification as well as other patient comorbidities (Table 1). Categorical variables were compared using the chi-squared test, and continuous variables were compared using Wilcoxon rank sum, Student’s t-test, analysis of variance or Kruskal–Wallis rank sum tests, depending upon distribution. Statistical significance was defined as *p* < 0.05. All analyses for this study were completed in SAS 9.4 (Cary, NC).

**Table 1:** Baseline demographic and clinical characteristics of all patients undergoing bariatric surgery between 2015 and 2021 and stratified by Black and White race (2015–2021)

Variables	Overall (*N* = 838,295)		Black (*N* = 193,071)		White (*N* = 645,224)		*p* Value
	Median	IQR	Median	IQR	Median	IQR	
Age, yrs	44.7	(36.0–53.8)	42	(34.4–50.5)	45.4	(36.5–54.7)	< 0.001
Body Mass Index (BMI), kg/m^2^	44	(40.0–49.0)	45	(41.0–51.0)	44	(40.0–49.0)	< 0.001

	*n*	%	*n*	%	*n*	%	
Sex							< 0.001
Female	674,993	80.52	168,453	87.25	506,540	78.51	
Male	163,260	19.48	24,606	12.74	138,654	21.49	
Non-binary	42	0.01	12	0.01	30	0	
Bariatric surgery performed							< 0.001
RYGB	229,965	27.43	40,637	21.05	189,328	29.34	
SG	608,330	72.57	152,434	78.95	455,896	70.66	
ASA class							< 0.001
1-No disturb	2194	0.26	592	0.31	1602	0.25	
2-Mild disturb	165,868	19.79	35,781	18.53	130,087	20.16	
3-Severe disturb	640,294	76.38	149,263	77.31	491,031	76.1	
4-Life threat	29,575	3.53	7350	3.81	22,225	3.44	
5-Moribund	64	0.01	17	0.01	47	0.01	
None assigned	300	0.04	68	0.04	232	0.04	
Pre-op functional health status							< 0.001
Independent	830,791	99.1	191,266	99.07	639,525	99.12	
Partially dependent	3882	0.46	952	0.49	2930	0.45	
Partially dependent	1107	0.13	308	0.16	799	0.12	
Totally dependent	2062	0.25	452	0.23	1610	0.25	
Totally dependent	33	0	11	0.01	22	0	
Unknown	420	0.05	82	0.04	338	0.05	
Pre-op hypertension	416,151	49.64	102,795	53.24	313,356	48.57	< 0.001
Pre-op hyperlipidemia	208,095	24.82	38,044	19.7	170,051	26.36	< 0.001
Pre-op renal insufficiency	5440	0.65	2006	1.04	3434	0.53	< 0.001
Pre-op diabetes mellitus	215,090	25.7	50,188	26.0	164,902	25.6	< 0.001
Pre-op obstructive sleep apnea	337,300	40.24	68,776	35.62	268,524	41.62	< 0.001
Pre-op history of COPD	14,662	1.75	2530	1.31	12,132	1.88	< 0.001
History of MI	11,354	1.35	1796	0.93	9558	1.48	< 0.001
Current smoker within 1 year	69,334	8.27	14,155	7.33	55,179	8.55	< 0.001
Pre-op steroid/immunosuppressant use for chronic condition	16,791	2	4003	2.07	12,788	1.98	0.012
Therapeutic anticoagulation	26,433	3.15	4628	2.4	21,805	3.38	< 0.001
Pre-op venous stasis	9063	1.08	1132	0.59	7931	1.23	< 0.001
Pre-op vein thrombosis requiring therapy	16,459	1.96	3349	1.73	13,110	2.03	< 0.001
History of PTE	12,287	1.47	2996	1.55	9291	1.44	< 0.001
Surgical approach							< 0.001
Robotic-assisted	56,622	6.75	14,147	7.33	42,475	6.58	

*IQR* InterQuartile Range, *ASA* American Society of Anesthesiologists, *Pre-op* preoperative, *MI* myocardial infarction, *COPD* chronic obstructive pulmonary disease, *PTE* pulmonary thromboembolism, *RYGB*: Roux-en-Y gastric bypass, *SG* sleeve gastrectomy

### Matched analyses

A matched case–control study was then performed. Black and White bariatric surgery patients were propensity-score matched one-to-one based on the following patient and surgical characteristics: age, BMI at time of operation, sex (male/female), operation performed (SG or RYGB), ASA classification, preoperative functional health status (independent/partially dependent), hypertension, hyperlipidemia, preoperative renal insufficiency, preoperative diabetes mellitus (DM), preoperative obstructive sleep apnea, preoperative history of chronic obstructive pulmonary disease (COPD), steroid/immunosuppressant use, therapeutic anticoagulation, and history of pulmonary thromboembolism (PTE). Perioperative outcomes were then compared by race (Black and White).

### Primary and secondary outcomes

The primary outcome was 30-day mortality following primary bariatric surgery. Secondary outcomes included 30-day hospital readmissions, hospital LOS, reoperations, and postoperative complications. Other postoperative complications included acute renal failure, PTE, intraoperative or postoperative cardiac arrest, stroke/cerebrovascular accident (CVA), progressive renal insufficiency, transfusion requirements within 72 h of surgery start time, wound disruption, surgical site infections, and postoperative pneumonia.

### Subgroup analyses

Among the matched patients, additional subgroup analyses were performed. The patients were stratified by procedure performed (SG or RYGB). Within these subgroups, primary and secondary outcomes were assessed by race (Black and White).

## Results

Overall, there were 193,071 Black and 645,224 White primary bariatric patients included in the initial study (Table 1). Black patients were younger [median age of 42 years; interquartile range (IQR) 34.4–50.5] than White patients (median age 45.4 years; IQR 36.5–54.7; *p* < 0.001). Black patients also had slightly higher median BMI of 45 kg/m^2^ (IQR 41–51), compared with median BMI of 44 kg/m^2^ (IQR 40–49) among Whites (*p* < 0.001). Black patients were more commonly female (87.25% vs. 78.51%; *p* < 0.001). Sleeve gastrectomy (SG) accounted for 78.95% of bariatric surgery among Black patients, compared with 70.66% of White patients (*p* < 0.001). Preoperative diagnoses of hypertension and renal insufficiency were more prevalent among Black patients, while White patients had higher rates of preoperative hyperlipidemia (Table 1). Prior history of smoking and history of MI was more commonly reported among White patients compared with their Black counterparts (Table 1). However, Black patients had higher reported histories of preoperative PTE (1.55% vs. 1.44%; *p* < 0.001).

### Matched case- control: overall

A total of 219,566 Black (*n* = 109,783) and White (*n* = 109,783) bariatric patients were matched and included in the case–control. The two groups were well matched (Table 2). Overall, Black patients were found to have higher rates of 30-day mortality compared with White patients (0.02% vs. 0.01%; *p* = 0.03) and readmission (3.68% vs. 2.65%; *p* < 0.001). The most commonly reported source for readmission was nausea/vomiting or abdominal pain for all bariatric patients. This was reported more often as the reason for readmission for Black patients (53.6% of readmissions vs. 43.4%; *p* < 0.0001). Additional causes of readmissions were similar between the groups, including risks of DVT/PE (about 8–9% of readmissions). There were also more Black patients readmitted for obstructions, internal hernias, or strictures (7.6% vs. 6.4%; *p* = 0.047), while more White patients had gastrointestinal leak or ulceration as the cause of readmission (8.3%, *n* = 242 vs. 3.2%, *n* = 129, *p* < 0.0001). Stroke readmissions accounted for 20 readmissions among Black patients and only 3 White patients. 368 Black patients were admitted for PTE/DVT, compared to only 237 White patients.

**Table 2:** Patient Characteristics with the Matched Case–Control Study, by race (comparing Black and White patients), (2015–2021)

Matched							
Variable	Overall (*N* = 219,566)		Black (*N* = 109,783)		White (*N* = 109,783)		*p* Value
	Median	IQR	Median	IQR	Median	IQR	
Age, yrs	40	(33.0–48.0)	40	(33.0–48.0)	40	(33.0–48.0)	1
Body mass index (BMI), kg/m^2^	43	(40.0–47.0)	43	(40.0–47.0)	43	(40.0–47.0)	1

	*n*	%	*n*	%	*n*	%	
Sex							1
Female	207,686	94.59	103,843	94.59	103,843	94.59	
Male	11,880	5.41	5940	5.41	5940	5.41	
Bariatric surgery performed							1
RYGB	38,332	17.46	19,166	17.46	19,166	17.46	
SG	181,234	82.54	90,617	82.54	90,617	82.54	
ASA class							1
1-no disturb	36	0.02	18	0.02	18	0.02	
2-mild disturb	43,294	19.72	21,647	19.72	21,647	19.72	
3-severe disturb	175,710	80.03	87,855	80.03	87,855	80.03	
4-life threat	510	0.23	255	0.23	255	0.23	
None assigned	16	0.01	8	0.01	8	0.01	
Pre-op functional health status, *n* (%)							1
Independent	219,556	100	109,778	100	109,778	100	
Partially dependent	10	0	5	0	5	0	
Pre-op hypertension	72,802	33.16	36,401	33.16	36,401	33.16	1
Pre-op hyperlipidemia	17,660	8.04	8830	8.04	8830	8.04	1
Pre-op renal insufficiency	8	0	4	0	4	0	1
Pre-op diabetes mellitus	17,492	7.97	8746	7.97	8746	7.97	1
Pre-op obstructive sleep apnea							1
	51,058	23.25	25,529	23.25	25,529	23.25	
Pre-op history of COPD	22	0.01	11	0.01	11	0.01	1
History of MI	14	0.01	7	0.01	7	0.01	1
Current smoker within 1 year	4708	2.14	2354	2.14	2354	2.14	1
Pre-op steroid/immunosuppressant use for chronic condition	256	0.12	128	0.12	128	0.12	1
Therapeutic anticoagulation	78	0.04	39	0.04	39	0.04	1
Pre-op venous stasis	14	0.01	7	0.01	7	0.01	1
Pre-op vein thrombosis requiring therapy	54	0.02	27	0.02	27	0.02	1
History of PTE	42	0.02	21	0.02	21	0.02	1
Surgical approach							1
Robotic-assisted	952	0.43	476	0.43	476	0.43	

*IQR* InterQuartile Range, *ASA* American Society of Anesthesiologists, *Pre-op* preoperative, *MI* myocardial infarction, *COPD* chronic obstructive pulmonary disease, *PTE* pulmonary thromboembolism, *RYGB* Roux-en-Y gastric bypass, *SG* sleeve gastrectomy

**Fig. 1:**
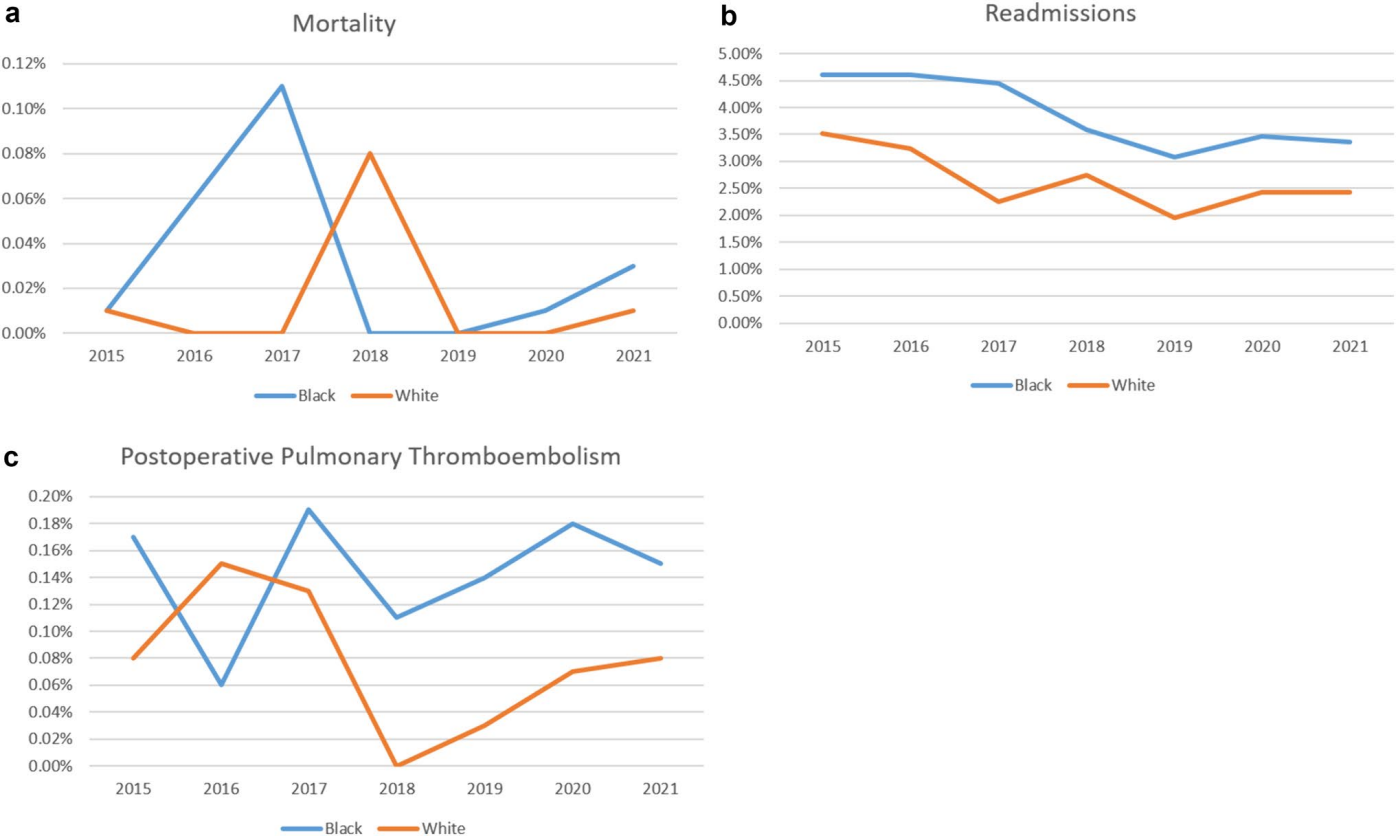
**a** Mortality rates for bariatric patients by race. **b** Readmissions for bariatric patients by race. **c** Postoperative Pulmonary Thromboembolism for bariatric patients by race

Median length of stay was 1.0 day (IQR 1.0–2.0) regardless of race, and there were no differences in reoperation rates (Table 3). However, there was a higher postoperative PTE rate for Blacks compared with Whites (0.16% vs. 0.08%; *p* < 0.001) (Table 3). There was also a higher rate of postoperative stroke/CVA among Black patients (0.02% vs. 0.01%; *p* = 0.01) (Table 3). Likewise, Black patients had higher rates of progressive renal insufficiency (0.05% vs. 0.02%; *p* < 0.001) (Table 3). The differences in perioperative outcomes stratified by race persisted over the study period (Fig. 1). White patients had higher rates of wound disruption and surgical site infections (Table 3).

**Table 3:** Postoperative outcomes among SG and RYGB patients, by race among matched case–control

Variable	Overall (*n* = 219,566)	Black (*n* = 109,783)	White (*n* = 109,783)	*p*-value
	*n* (%)	*n* (%)	*n* (%)	
30-day mortality	33 (0.02)	24 (0.02)	9 (0.01)	0.03
30-day readmissions	6948 (3.16)	4044 (3.68)	2904 (2.65)	< 0.001
Hospital length of stay (LOS) median (IQR)	1 (1.0–2.0)	1 (1.0–2.0)	1 (1.0–2.0)	< 0.001
Any complication	3804 (1.73)	1824 (1.66)	1980 (1.8)	0.01
30-day reoperations	2060 (0.94)	1052 (0.96)	1008 (0.92)	0.33
Acute renal failure	61 (0.03)	34 (0.03)	27 (0.02)	0.37
Intra-op or post-op cardiac arrest requiring CPR	30 (0.01)	18 (0.02)	12 (0.01)	0.27
Stroke/CVA	29 (0.01)	22 (0.02)	7 (0.01)	0.01
Intra-op or post-op myocardial infarction	27 (0.01)	14 (0.01)	13 (0.01)	0.85
Progressive renal insufficiency	85 (0.04)	60 (0.05)	25 (0.02)	< 0.001
Pulmonary thromboembolism	259 (0.12)	174 (0.16)	85 (0.08)	< 0.001
Transfusion intra-op/post-op (72 h of surgery start time)	1185 (0.54)	622 (0.57)	563 (0.51)	0.09
Wound disruption	53 (0.02)	11 (0.01)	42 (0.04)	< 0.001
Post op superficial SSI	599 (0.27)	213 (0.19)	386 (0.35)	< 0.001
Postop deep SSI	75 (0.03)	24 (0.02)	51 (0.05)	0.002
Postop pneumonia	531 (0.24)	221 (0.20)	310 (0.28)	0.40

*Intra-op* Intraoperative, *Post-op* postoperative, *CVA* cardiovascular accident, *SSI* surgical site infections

### Subgroup analyses

#### Matched SG

Within the matched case–control, a total of 181,234 patients underwent SG, including 90,617 Black and White patients. Among SG patients, Black patients were again found to have higher rates of 30-day mortality and 30-day readmission (Table 4). Median LOS remained at 1 day for both Black and White patients. Black patients were also found to have higher incidences of progressive renal insufficiency and PTE (Table 4). Black SG patients also had higher rates of perioperative or postoperative transfusion requirements (0.51% vs. 0.44%; *p* = 0.03). White patients continued to have higher surgical site infections in the SG cohort (Table 4).

**Table 4:** Postoperative outcomes among SG patients, by race among matched case–control

Variable	Overall (*n* = 181,234)	Black (*n* = 90,617)	White (*n* = 90,617)	*p*-value
	*n* (%)	*n* (%)	*n* (%)	
30-day mortality	30 (0.02)	21 (0.02)	9 (0.01)	0.03
30-day readmissions	4970 (2.74)	2999 (3.31)	1971 (2.18)	< 0.001
Hospital length of stay (LOS), median (IQR)	1 (1.0–2.0)	1 (1.0–2.0)	1 (1.0–2.0)	< 0.001
Any complication	2649 (1.46)	1278 (1.41)	1371 (1.51)	0.07
30-day reoperations	1275 (0.70)	654 (0.72)	621 (0.69)	0.35
Acute renal failure	33 (0.02)	12 (0.01)	21 (0.02)	0.12
Intra-op or post-op cardiac arrest requiring CPR	22 (0.1)	10 (0.01)	12 (0.01)	0.67
Stroke/CVA	11 (0.01)	6 (0.01)	5 (0.01)	0.76
Intra-op or post-op myocardial infarction	24 (0.01)	11 (0.01)	13 (0.01)	0.68
Progressive renal insufficiency	76 (0.04)	56 (0.06)	20 (0.02)	< 0.001
Pulmonary thromboembolism	175 (0.10)	111 (0.12)	64 (0.07)	< 0.001
Transfusion intra-op/post-Op (72 h of surgery start time)	861 (0.48)	463 (0.51)	398 (0.44)	0.03
Wound disruption	40 (0.02)	11 (0.01)	29 (0.03)	0.004
Post op superficial SSI	397 (0.22)	127 (0.14)	270 (0.30)	< 0.001
Postop deep SSI	37 (0.02)	15 (0.02)	22 (0.02)	0.25
Postop pneumonia	140 (0.08)	61 (0.07)	79 (0.09)	0.13

*Intra-op* Intraoperative, *Post-op* postoperative, *CVA* cardiovascular accident, *SSI* surgical site infections

#### Matched RYGB

Among the matched patients, 38,332 underwent RYGB (19,166 Black and White patients). There were no statistically significant differences in postoperative mortality among RYGB patients by race, but a higher readmission rate was found for Black patients (5.45% vs. 4.87%; *p* = 0.01). Compared with White RYGB patients, Black RYGB patients also had longer median LOS (2.0 vs. 1.0 day; *p* < 0.001) and higher rates of acute renal failure (0.11% vs. 0.03%; *p* = 0.002). Black patients who underwent RYGB were found to have higher rates of stroke/CVA, intraoperative or postoperative cardiac arrest requiring cardiopulmonary resuscitation and PTEs (Table 5). White patients continued to have higher rates of surgical site infections among RYGB patients (Table 5).

**Table 5:** Postoperative Outcomes among RYGB patients, by Race among matched case–control

Variable	Overall (*n* = 38,332)	Black (*n* = 19,166)	White (*n* = 19,166)	*p*-value 0.08
	*n* (%)	*n* (%)	*n* (%)	
30-day mortality	3 (0.01)	3 (0.02)	0 (0)	
30-day Readmissions	1978 (5.16)	1045 (5.45)	933 (4.87)	0.01
Hospital length of stay (LOS), median (IQR)	1 (1.0–2.0)	2 (1.0–2.0)	1 (1.0–2.0)	< 0.001
Any complication	1155 (3.01)	546 (2.85)	609 (3.18)	0.06
30-day reoperations	785 (2.05)	398 (2.08)	387 (2.02)	0.69
Acute renal failure	28 (0.07)	22 (0.11)	6 (0.03)	0.002
Intra-op or post-op cardiac arrest requiring CPR	8 (0.02)	8 (0.02)	0 (0)	0.005
Stroke/CVA	18 (0.05)	16 (0.08)	2 (0.01)	< 0.001
Intra-op or post-op myocardial infarction	3 (0.01)	3 (0.01)	0 (0)	0.08
Progressive renal insufficiency	9 (0.02)	4 (0.02)	5 (0.03)	0.74
Pulmonary thromboembolism	84 (0.22)	63 (0.33)	21 (0.11)	< 0.001
Transfusion intra-op/post-op (72 h of surgery start time)	324 (0.85)	159 (0.83)	165 (0.86)	0.74
Wound disruption	13 (0.03)	0 (0)	13 (0.07)	< 0.001
Post op superficial SSI	202 (0.53)	86 (0.45)	116 (0.61)	0.03
Postop deep SSI	38 (0.10)	9 (0.05)	29 (0.15)	0.001
Postop pneumonia	95 (0.25)	50 (0.26)	45 (0.23)	0.61

*Intra-op* Intraoperative, *Post-op* postoperative, *CVA* cardiovascular accident, *SSI* surgical site infections

## Discussion

In this study of MBSAQIP data from 2015 through 2021, Black patients accounted for < 25% of primary bariatric surgery performed in the United States, despite the known higher prevalence of obesity among Black patients. Within the overall propensity score matched case–control, Black patients were found to have higher rates of perioperative mortality and hospital readmissions following bariatric surgery. Black patients also had higher rates of other perioperative complications including PTE, CVA, and progressive renal insufficiency following bariatric surgery. Within the subgroup analyses by procedure performed, Black patients undergoing SG had higher rates of postoperative blood transfusion requirements than White patients. Additionally, among patients who underwent RYGB, Black patients had longer LOS and higher rates of acute renal failure, stroke, and cardiac arrest.

The results of this study are consistent with previously published studies from the last decade, with continued evidence of low rates of complications following bariatric surgery [15, 16]. However, this study also demonstrates that worse perioperative outcomes persist for Black bariatric patients, compared with White bariatric patients. Despite the previous identification of racial disparities in postoperative outcomes for Black patients [13, 14, 17, 18], it remains largely unknown which factor(s) are contributing to the observed differences in postoperative outcomes for racial minority patients.

Socioeconomic factors have been demonstrated to be barriers to bariatric surgery [19]. Nonetheless, even with access to bariatric surgical care, racial minority patients continue to have worse postoperative outcomes. While this national study was not able to account for socioecological factors that may also contribute to these disparities, the identification of persistent disparities highlights the need to promote equity in healthcare for bariatric patients. Interestingly, Liu et al. found that Medicaid status and other social determinants of health at the neighborhood level were not associated with differences in long term outcomes such as weight loss at one year following bariatric surgery [20]. This group also found that patient-centered factors were more closely associated with 1-year outcomes than neighborhood level factors [21]. However, the relationship between these patient and community-level factors at the national level and short-term outcomes following bariatric surgery remains unclear.

Social determinants of health, including poor health literacy, have also been shown to impact access to bariatric surgery and outcomes following surgery. In their single institution study, Mahoney et al. found that lower health literacy levels were associated with worse long-term outcomes, including less weight loss following bariatric surgery [22]. The authors also previously published that patients with educational levels less than high school graduation had three-fold increased risks of hospital visits following bariatric surgery [22]. Furthermore, lower health literacy levels have been historically found among racial minority patients, further exacerbating inequalities in access to quality surgical care [23]. While lower health literacy may not directly account for higher rates of mortality for Black patients in this study, health literacy may play a role in the observed increased readmission rates for Black patients following bariatric surgery. As many of the readmissions, particularly among Black patients, were due to nausea/vomiting and abdominal pain, there may be opportunities for improvement in both the inpatient and outpatient settings to enhance symptom control and management. Importantly, this also highlights an actionable opportunity for patient-centered interventions, such as patient navigation systems [24], in order to provide more resources and bridge the health literacy gap for vulnerable patient populations. Likewise, improved access to primary care for racial minority patients may lead to better preoperative screenings and diagnoses of cardiovascular disease, which may also improve disparities in postoperative outcomes. However, future prospective studies are also needed among racially diverse bariatric patient populations in order to further identify barriers to equity in perioperative and surgical care so that they may be ameliorated.

This study is not without limitations. Due to the nature of the national dataset provided by the MBSAQIP, there is a lack of granularity to help elucidate the factors that may be contributing to worse outcomes in Black patients. For example, socioecological factors that may contribute to the observed findings are not reported by MBSAQIP. There may also be inconsistencies in reporting and capturing perioperative information as presented by the MBSAQIP database. Additionally, the data is limited to programs participating in the MBSAQIP accreditation and therefore may not be representative of the national population as a whole. Despite attempting to mitigate confounding bias in the study through propensity score matching, it is possible that residual confounding variables exist. The data also does not include information regarding patients’ access to primary care, which may influence postoperative outcomes, particularly those related to perioperative cardiovascular risks. Similarly, the study is not able to account for underlying differences such as under-treatment or under-diagnosis of hypertension, or renal disease which may be more prevalent among Black patients. Although prior work has demonstrated a relationship between health literacy and perioperative outcomes, unfortunately this study was not able to account for patients’ health literacy. However, we have several on-going studies that are investigating this knowledge gap. Finally, the study was limited to primary SG and RYGB patients and therefore does not account for the outcomes following other metabolic and bariatric operations or revisional surgery.

In summary, this national study demonstrated that Black primary bariatric surgery patients in the United States continue to have higher mortality and readmission rates and worse perioperative outcomes than their White counterparts. Socioecological factors may be driving these observed disparities in perioperative outcomes. Further work must be done to in order to determine contributing factors and effect improvement in outcomes in metabolic and bariatric surgical care for racial minority patients.
